# Advancements and strategies of genetic improvement in cassava (*Manihot esculenta* Crantz): from conventional to genomic approaches

**DOI:** 10.1093/hr/uhae341

**Published:** 2024-12-02

**Authors:** Liang Xiao, Dong Cheng, Wenjun Ou, Xin Chen, Ismail Yusuf Rabbi, Wenquan Wang, Kaimian Li, Huabing Yan

**Affiliations:** Cash Crops Research Institute, Guangxi Academy of Agricultural Sciences, Nanning 530007, China; Cash Crops Research Institute, Guangxi Academy of Agricultural Sciences, Nanning 530007, China; Tropical Crop Genetic Resources Institute, Chinese Academy of Tropical Agricultural Sciences, Haikou 571101, China; Institute of Tropical Bioscience and Biotechnology, Chinese Academy of Tropical Agricultural Sciences, Haikou 571101, China; International Institute of Tropical Agriculture, Ibadan 200001, Nigeria; National Key Laboratory of Biotechnology and Breeding of Tropical Crops, Hainan University, Haikou 570228, China; Tropical Crop Genetic Resources Institute, Chinese Academy of Tropical Agricultural Sciences, Haikou 571101, China; Cash Crops Research Institute, Guangxi Academy of Agricultural Sciences, Nanning 530007, China

## Abstract

Cassava (*Manihot esculenta* Crantz) is a staple food of 800 million people in the tropical and subtropical regions of the world. Its industrial utilization for bioethanol, animal feed, and starch are still continuously expanding. It was not until the 1970s that significant scientific efforts were undertaken to improve cassava, despite its considerable economic and social significance. Shortening the breeding cycle and improving the breeding efficiency are always the focus of the cassava breeding study. In this review, we provide a global perspective on the current status of cassava germplasm resources and explore the diverse applications of cassava breeding methods from hybridization, polyploidy, and inbreeding to genomic selection and gene editing. Additionally, we overview at least six nearly complete cassava genome sequences established based on modern genomic techniques. These achievements have substantially supported the advancing of gene discovery and breeding of new cassava varieties. Furthermore, we provide a summary of the advancements in cassava’s functional genomics, concentrating on important traits such as starch quality and content, dry matter content, tolerance to postharvest physiological deterioration, nutritional quality, and stress resistance. We also provide a comprehensive summary of the milestone events and key advancements in cassava genetic improvement over the past 50 years. Finally, we put forward the perspective of developing genomic selection breeding model and super-hybrids of cassava through building inbreeding population and emphasize the generation of triploid cassavas, as well as using gene editing technology allowing cassava to be a tropical model plant to serve for basic biological research and molecular breeding.

## Introduction

Cassava (*Manihot esculenta* Crantz) (2n = 36), is an important tuber crop cultivated in tropical and subtropical regions in the world. A variety of African countries, with Nigeria at the forefront, are prominent in production, including the Democratic Republic of the Congo, Ghana, Angola, Tanzania, and Cameroon. In Asia, Thailand, Indonesia, Vietnam, Cambodia, China, and India are also key producers. Furthermore, Brazil and Paraguay are significant producers in Latin America [1]. According to FAOSTAT, the global production of cassava in 2022 exceeded 300 million tons, spanning >30 million hectares. Africa accounted for a significant portion of this output, contributing 63.3%, while Asia contributed 29.1%. Originating from South American countries, cassava belonging to the Euphorbiaceae family, genus *Manihot*, was domesticated by indigenous Amazonian peoples >9000–12 000 years ago through the cultivation of its wild ancestor, *M. esculenta* ssp. *flabellifolia* [2]. After being brought to Africa by traders in the 16th century, cassava spread rapidly throughout tropical areas, especially in sub-Saharan Africa, India, and Southeast Asia. Today, cassava serves as a staple food for nearly 1 billion people across 105 countries in Africa, South America, and Asia. There is also a growing need for its byproducts, including starch, animal feed, and bioethanol [3]. Cassava is well adapted to drought and low-fertility soils that are prevalent in vast tropical regions and demonstrates remarkable resilience to the adverse conditions of low soil pH and high aluminum saturation, which are often encountered in such soils. Cassava is capable of yielding substantial harvests in conditions where many other crops would fail due to the acidic pH and elevated aluminum levels [4, 5].

Despite its significance, cassava breeding has historically encountered considerable challenges due to its high heterozygosity, difficult and asynchronous flowering, and limited seed production. The high degree of genomic heterozygosity is the general reason for the low efficiency of conventional hybridization breeding, just as happened in corn [6]. However, in recent decades, innovative plant breeding methods have been devised to overcome these obstacles. These methods encompass the evaluation and selection of indigenous landraces, introduction of novel germplasm, hybridization techniques, polyploid breeding, and genetic transformation. Pioneering breeding programs, led by the International Center for Tropical Agriculture (CIAT) and the International Institute of Tropical Agriculture (IITA), have spearheaded successful breeding projects, such as inbreeding, resulting in the cultivation of superior cassava varieties with enhanced productivity or valued quality and stress resistance. Nonetheless, cassava breeding continues to confront challenges, such as establishing protocols for homozygous lines production, and integrating molecular marker technology into practical breeding applications [7]. The emergence of next-generation sequencing technologies has dramatically transformed the landscape of plant genomics, offering new opportunities for understanding the complex genome of cassava. The sequencing of the cassava genome has provided valuable insights into its genetic diversity, evolutionary history, and the identification of genes associated with important agronomic traits [8]. Advanced biotechnological techniques have further enabled the assembly of more accurate and complete genome sequences. These advancements are pivotal for detecting structural variations (SVs), single-nucleotide variations (SNVs), and phasing haplotypes [9], thereby deepening our comprehension of cassava’s genetic architecture. In summary, the current status of cassava breeding is marked by a combination of traditional and modern breeding techniques, supported by the wealth of genomic information. The ongoing research and development in cassava genomics are poised to unlock the full potential of this crop, leading to the creation of improved varieties that can better withstand environmental challenges and meet the nutritional needs of millions of people.

**Figure 1:**
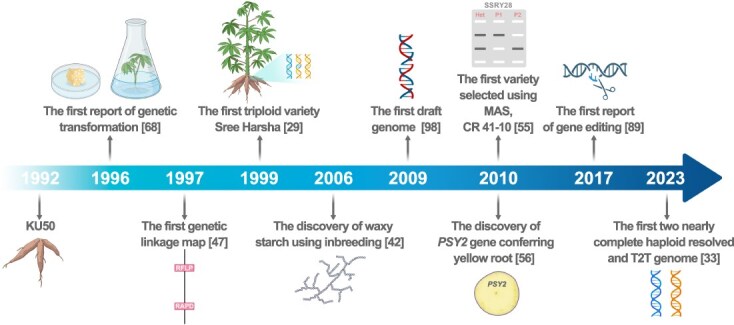
The milestone events and key advances of cassava genetic improvement over the past 50 years

In this review, we present an overview of the current status of cassava breeding methods and stress on foundation in reference genomes and pan-genome and progress of genetic improvement of universal traits in cassava. Moreover, we provide a comprehensive summary of the milestone events and key advancements in cassava genetic improvement over the past 50 years (Fig. 1). Furthermore, we highlight the significant breeding challenges that require our attention, stemming from the impacts of climate change and the growing demand for the diversification of cassava’s applications.

## Cassava germplasm resources

Genetic enhancement is initiated through the collection and assessment of a broad spectrum of germplasm resources. The *Manihot* genus encompasses 98 species, with *M. esculenta* being the only cultivated one. Among these 98 species, 81 are distributed in Brazil, and 17 in Mexico. The global collection of cassava germplasm resources amounts to 13 832 samples, primarily concentrated in nine countries across South America, Central Africa, West Africa, and Southeast Asia. The main authoritative institutions engaged in the preservation, evaluation, and innovative utilization research of cassava germplasm resources are International Bioversity Center (IBC)-CIAT, Brazilian Agricultural Research Corporation (EMBRAPA), and IITA. CIAT possesses the world’s largest cassava germplasm bank, collecting and preserving 5963 resources from 141 countries worldwide (with 5577 cultivated and 386 wild resources), leading in terms of resource preservation. IITA holds >2000 samples, while EMBRAPA preserves 3810. Additionally, the Chinese Academy of Tropical Agricultural Sciences (CATAS) maintains >3000 samples, and the Guangxi Academy of Agricultural Sciences (GXAAS) preserves >4000 samples (Personal communication). Resource-rich countries such as Brazil and Colombia have established national germplasm resource management systems to strengthen the preservation, evaluation, and sharing of technology systems for germplasm resources. However, many tropical and subtropical regions of the world are developing countries, where the investment and research levels in tropical crop science and technology are generally low. Although a large amount of cassava germplasm resources has been collected, there are issues such as unclear background information, lack of key trait genes, and low breeding efficiency. As a result, the cassava germplasm resources for key traits have not been fully explored.

## Methods for cassava breeding

The field of cassava breeding has seen the emergence and maturation of diverse methods aimed at enhancing its traits. In this context, we outline the advancements in nine breeding methods for cassava, including conventional breeding, interspecific hybridization, polyploidy, mutation breeding, inbreeding, maker-assisted selection (MAS), genomic selection (GS), genetic transformation, and gene editing.

### Conventional breeding

The process of evaluating and selecting both introduced and local landraces is a cornerstone in the realm of cassava breeding. A significant proportion of cassava cultivars released in Asia have been meticulously chosen from these introduced varieties or F1 seeds, such as in Thailand and China, highlighting the crucial role of this approach in enhancing the genetic diversity of the crop. Over recent years, a substantial quantity of accessions from CIAT’s cassava gene bank, along with hybrid F1 seeds from CIAT’s breeding programs, have been annually dispatched to countries across the Americas, Africa, and Asia, reinforcing the global exchange and utilization of cassava genetic resources [10]. Hybridization, another pivotal breeding method, entails the crossbreeding of distinct clones within the species to forge novel gene combinations. This technique is commonly applied in mass phenotypic recurrent selection, where breeders identify the top-performing individuals based on observable traits and subsequently utilize them in the subsequent breeding cycle. Recurrent selection is a method designed to augment the prevalence of advantageous alleles within a population through a cyclical process of selection and intercrossing of individuals exhibiting favorable characteristics. By engaging in several cycles of selection, assessment, and genetic recombination, recurrent selection effectively leverages genetic variability to secure sustainable genetic advancements across generations. The breeding cycle of mass phenotypic recurrent selection begins with the production of full- or half-sib seed in crossing blocks, followed by the selection of high-heritability traits in seedling nurseries, such as disease resistance, high carotenoid content, amylose-free (waxy) starch. Selected individual cassava plants are then cloned for progression through single-row trials, preliminary yield trials, advanced yield trials, and ultimately uniform yield trials, which span several years [11]. Parents for the next generation are selected from the late testing stages. This method has been successful in developing outstanding cassava varieties, such as KU50 released in 1992 (Fig. 1), which is still widely grown in Southeast Asia. Two major breeding programs are operational in Thailand: Kasetsart University (which has released KU50, HB60, HB80, and HB90) and Rayong Field Crops Research Center, which released excellent varieties such as Rayong 5, Rayong 7, Rayong 9, Rayong 72, and Rayong 90.

A considerable challenge in cassava breeding through recurrent selection is the variability in the reproductive timing among different genotypes, which complicates the synchronization of flowering dates for cross-pollination. Some clones commence flowering early, while others flower relatively late, and a few may not flower at all. Particularly, the elite cultivar characterized by its erect plant architecture tends to flower infrequently or not at all in certain regions. The recent advancements in flower-inducing technologies offer a viable solution to this challenge. Ethylene signaling plays a crucial role in cassava floral development, and the antiethylene plant growth regulator silver thiosulfate can counteract the effects of ethylene on this process [12]. Moreover, the implementation of flower-inducing techniques, extending photoperiod (red lights), and relative cool day temperature (~22°C), has been shown to stimulate flowering in cassava, thus enabling controlled crosses and the production of seeds for breeding purposes [13, 14]. This approach can significantly shorten the breeding cycle and increase the efficiency of developing new varieties with improved traits.

### Interspecific hybridization

Interspecific hybridization plays a critical role in enhancing cassava breeding. *Manihot fabellifolia* and *Manihot peruviana* are recognized as the principal gene pools for domesticated cassava [15]. Wild *Manihot* germplasm serves as a treasure trove of beneficial genes for the cultivated *M. esculenta* species, offering traits such as amylose-free or low-amylose-content starch found in *Manihot chlorostricta* and *Manihot crassisepala* [16], and high protein levels observed in *M. peruviana*, *M. flabellifolia*, and *Manihot tristis* [17]. Notable successes in interspecific hybridization include UnB 110, a high-yielding, drought-resistant clone derived from a cross with *Manihot glaziovii*, which also showed immunity to the cassava mealybug [18]. Especially, the breeding program in Tanzania effectively utilized *M. glaziovii* to address cassava mosaic disease (CMD), a heavy threat to production. The hybrids gifted by Prof. Nagib Nassar (University of Brasilia), who successfully created >20 species-wide hybrids include of the progeny from *M. glaziovii*, that being the only resistance of CMD up to now. The early 20th-century efforts in Tanzania led to the development of CMD-resistant varieties, transferring genes from *M. glaziovii* to enhance disease resistance of the local cultivars while maintaining root quality. The hybrid clone 58 308 was identified as CMD-resistant in 1958; it showed continuous resistance for 20 years, but its yield was very poor [19]. Moderate to high levels of resistance to cassava green mite (CGM), whitefly, and the cassava mealybug have been identified in interspecific hybrids of *M. flabellifolia* [16]. Furthermore, interspecific hybridization can break through reproductive isolation to obtain interspecific or even intergeneric hybrids with apomixis, allowing for the preservation of hybrid vigor across generations and the popularization and utilization of new cassava varieties through asexual reproduction [20]. Despite the potential valuable traits of wild *Manihot* species, their integration into regular breeding programs is hindered by low fertility, linkage drag, and the extended reproductive breeding cycle, often spanning 10–15 years.

### Polyploidy

Polyploidy, or whole-genome duplication (WGD), plays a pivotal role in the emergence of new plant species and plant evolution and breeding, despite potential weaknesses (such as sterility and unstable genome) [21]. Polyploid plants are often characterized by enhanced biological potential, improved environmental adaptability, increased biomass, and higher yield [22–24]. Polyploidy breeding has demonstrated unique advantages in cassava. UnB 201, an autotetraploid cassava variety, is distinguished by its high protein content [5.5% by dry weight (DW)], very low cyanogenic glucoside (CG) levels (~10–12 mg/kg of fresh root weight), and elevated β-carotene content reaching 27 mg/kg [25]. Autotetraploid cassava exhibits larger and thicker leaves, reduced plant height, and enhanced drought resistance compared to its diploid parent [26, 27]. Notably, triploid cassava has emerged as a cultivar with a suite of beneficial traits that enhance both agricultural productivity and culinary appeal. These plants exhibit higher yields, increased dry matter and starch content in their roots, an elevated harvest index (HI), accelerated bulking, and the ability to be harvested early. Additionally, they demonstrate resistance to CMD and commendable shade tolerance. This triploid form of cassava not only offers high yields but also stands out for its culinary quality, making it a favored choice for a range of applications, from industrial processing to household use [28]. The first triploid cassava variety, Sree Harsha (Fig. 1), developed by crossing cultivated diploids with colchicine-induced tetraploids, is renowned for its robust growth, upright plant structure, broad leaves, and stout stems [29]. Furthermore, two additional triploid hybrids, Sree Athulya and Sree Apporva, are celebrated for their high extractable starch content, which exceeds 30% [30]. Our research team has also observed remarkable characteristics in a triploid cassava line derived from the cross of the parent line Xinxuan 048 with its autotetraploid. This line has demonstrated remarkable characteristics by exhibiting the highest photosynthetic capacity, achieving the most substantial yield, possessing the largest aboveground biomass, and bearing the heaviest tuber weight per plant. It also stands out with the highest HI and the greatest number of tubers produced per plant among the three cytotypes (Unpublished data). These findings underscore the potential of triploid cassava in advancing agricultural outputs and diversifying the uses of this versatile crop. It must be mentioned that cassava is a typical asexual reproduction crop, and the excellent polyploid varieties obtained could be stably inherited through asexual reproduction and applied in production.

### Mutation breeding

Mutagenesis in cassava, a crop known for its random nature, can lead to both beneficial and detrimental outcomes. Despite the unpredictability, a few notable successes have been documented, excluding those involving polyploids induced by colchicine. Two mutant cassava varieties have been documented in the International Atomic Energy Agency (IAEA) database (http://mvgs.iaea.org/). One variety, Tebankye, is notable for its large starch granules in the tubers and resistance to CMD. Another distinguished cassava clone, Fuxuan 01, was selected from a mutated population using SC124 as the progenitor and released in China in 2005. CIAT has identified several exceptional clones with small granules and high-amylose starch, as well as traits like tolerance to postharvest physiological deterioration (PPD), ‘hollow’ starch granules, or even starchless characteristics, from an M2 population derived from 1400 seeds that were irradiated in their true form [31].

TILLING (Targeting Induced Local Lesions IN Genomes) does not differ from conventional mutation breeding in terms of the organisms involved, thus avoiding the complexities associated with genetically modified organisms. This characteristic makes TILLING a compelling approach for both functional genomics and agricultural applications [32]. The technique offers several advantages over traditional methods of crop enhancement. Firstly, it generates a variety of allelic mutations that are advantageous for genetic studies. Secondly, mutations that are difficult to identify through forward genetics can be detected using TILLING, as it concentrates on the gene of interest. Thirdly, it is universally applicable to any organism. With the recent public release of the cassava genome assembly, particularly the comprehensive telomere-to-telomere (T2T) reference genome sequence [33], detailed gene annotations and allelic diversity information essential for TILLING have become accessible. EcoTILLING, a variation of TILLING, is utilized to detect rare single-nucleotide polymorphism (SNPs) or small INDELs in target genes within natural populations [34]. Although applications of TILLING or EcoTILLING for genetic improvement are limited, efforts to irradiate seeds of elite cassava lines and wild *Manihot* species have aimed to diversify the genetic base and broaden industrial applications [35].

Given that cassava primarily relies on vegetative propagation, mutation breeding in this crop faces the challenges of chimerism and the lack of meiosis, which are essential for eliminating harmful alleles and revealing recessive genes [36]. Despite these obstacles, innovative strategies have been crafted to counteract these limitations. A notable example is the successful application of the TILLING/EcoTILLG protocol, combined with the multiapexing technique to eliminate chimerism, and traditional induction methods in the mutation breeding of bananas (*Musa* spp.) [37]. Drawing inspiration from this, the multiapexing technique could be adapted for cassava mutation breeding, employing *in vivo* stakes or *in vitro* stems as explants [38]. This method could effectively address the issue of chimerism, a common hurdle in the propagation of genetically modified plants. One of the significant hurdles in banana mutation breeding is the difficulty in identifying recessive loss-of-function phenotypes due to the sterility of triploid bananas. This challenge, however, is not an impediment in cassava. Self-hybridization in cassava provides a viable solution, allowing for the detection of recessive mutations. Since genetic modifications can be discerned using TILLING/EcoTILLG in the M1 generation, cassava breeders can proceed with propagation, field planting, and self-hybridization before conducting phenotypic evaluations. This streamlined approach facilitates a more efficient and targeted mutation breeding process in cassava, paving the way for the development of improved varieties that can withstand various agricultural challenges.

### Inbreeding

Cassava is predominantly heterozygous, and inbreeding has been minimally practiced in its breeding history. The introgression of traits in cassava faces several challenges that could be effectively addressed with the use of inbred lines. The frequency of deleterious alleles has increased as a result of clonal propagation [39]. However, current breeding methods have proven to be ineffective in purging the burden of deleterious alleles. Incorporating inbreeding into cassava genetic enhancement presents numerous advantages [7]. Inbreeding make sense for breeding as: (1) Inbreeding can help in reducing the genetic load. In contrast to traditional breeding techniques that mask deleterious mutations by maintaining them in a heterozygous state, inbred-parent-based hybrid breeding actively eliminates harmful genetic factors. This meticulous process ensures that the resulting hybrids are endowed with a purer genetic profile, which in turn enhances their overall vitality, productivity, and stability, as well as adaptability of the hybrid cassava cultivars. (2) Inbreeding can lead to the development of superior hybrids by design. Near-pure lines are theoretically obtained by inbreeding 4–5 rounds, which enable creation of super hybrids by crosses between alien pure lines, a method that has been used in corn normally [6]. (3) Inbreeding can facilitate obtaining new economic traits. Inbred lines of cassava can help identify additional desirable recessive alleles. Some beneficial recessive traits, such as small granules, high amylose, and waxy starch have been developed through inbreeding [40, 41]. The development of waxy starch in cassava is a prime example [40] (Fig. 1), offering significant advantages for functional properties and potential applications, leading to the starch industry’s investment in commercial varieties producing this type of starch. Another notable example is the identification of a petioless phenotype in an S1 family, which could improve the quality of foliage for animal feeding due to reduced fiber content from petioles [42]. (4) Inbreeding can simplify superior clone maintenance. Superior hybrids are often vegetatively propagated plants, which can lead to contamination by pathogens if not managed properly. Inbreeding allows for the easy rejuvenation of planting material through recrossing, a process that is more cost-effective and faster than the traditional tissue culture protocols. (5) Inbreeding can enhance genetic research. Inbreeding population can be used for discovery of genes for the high heterozygotic cassava genomes; it seems that inbreeding population is better than hybrid population and nature population for effective gene mapping. The availability of homozygous progenitors would greatly simplify the logistics of genetic research, allowing for higher contrast and ‘cleaner’ segregations in segregating progenies. This would also enable the identification of a larger number of recessive genes, both beneficial and detrimental, thus deepening a better understanding of genetic segregations. (6) Inbreeding can shorten breeding cycles. Elite heterozygous progenitors are typically crossed to generate full-sib families, with each F1 seed being genetically unique. Given cassava’s low multiplication rate, it takes 4–5 years to produce sufficient planting material for multilocation trials. Inbreeding parents would allow for multiple pollinations between the same progenitors, yielding genetically identical F1 hybrids, effectively reducing the evaluation cycle by 2 or 3 years. On the other hand, it is a common observation that crops exhibiting pronounced heterotic effects also experience notable inbreeding depression. These two biological phenomena (inbreeding depression and heterosis) are likely to be two sides of a coin and exist among a wide range of plant species [43, 44]. In S1 populations, inbreeding depression has been noted to affect aboveground yield, leaf diseases severity, as well as fresh root yield (FRY), primarily due to the significant role of additive genetic effects [7]. Building on the theory and technology of whole-genome design breeding of hybrid potato (*Solanum tuberosum*) [45, 46], we can resequence the S1 population and employ whole-genome-based segregation analysis along with phenotypic evaluation, and then pinpoint harmful mutations that significantly impact inbreeding depression in cassava. Breaking the linkage between these harmful mutations and desirable alleles allows us to clarify the genetic mechanisms that underline inbreeding depression in cassava.

Despite these advantages, the practical application of inbreeding in cassava presents challenges. Producing fully homozygous genotypes through successive self-pollinations takes 12–15 years, given that each self-pollination cycle requires a minimum of 2 years. Additionally, inbreeding may inadvertently promote early-flowering cassava genotypes, which may not necessarily correlate with robust growth. Many high-yielding and vigorous cassava varieties are naturally nonflowering, and it is these types that exhibit the erect plant architecture favored by farmers. The selective pressure to produce flowering lineages can inadvertently result in the development of cassava clones with less desirable plant architectural traits.

### Marker-assisted selection

In 1997, the first genetic linkage map of cassava was constructed (Fig. 1), incorporating 132 RFLPs, 30 RAPDs, three SSRs, and three isoenzyme markers, all of which were inherited from the heterozygous female parent in an intraspecific cross [47]. Despite the classical MAS employing these markers, its influence on cassava improvement has been somewhat restricted. However, the advent of next-generation sequencing technologies, particularly those utilizing SNP markers, holds promise to surmount many of the challenges associated with traditional MAS. Nowadays, large numbers of SNPs can be discerned with remarkable precision across the genome at an affordable cost. For instance, recently, Rabbi *et al.* [48] conducted a comprehensive genome-wide association study (GWAS), utilizing a diverse array of 5130 clones to identify quantitative trait loci (QTLs) correlated with 14 distinct traits, encompassing quality, stress response, and agricultural morphology. The study’s population was meticulously genotyped at an impressive 101 521 SNP markers through the innovative technique of genotyping-by-sequencing (GBS). Furthermore, a comprehensive set of 1 155 988 high-confidence SNPs was employed in the metabolic GWAS (mGWAS) to elucidate the genetic underpinnings of metabolites within the cassava storage root. The findings revealed that the *MeMYB4* gene is pivotal in governing the variability of cyanidin 3-O-glucoside and delphinidin 3-O-rutinoside levels, thereby dictating the color of the storage root endothelium [49]. Over the past two decades, substantial efforts have been directed toward identifying molecular markers linked to CG content [50], the height of plant and first branch [51], starch content [52], β-carotene content [53], resistance against cassava bacterial blight (CBB) [54], CMD, cassava brown streak disease (CBSD), CGM [48], etc (See the section of ‘Gene mining of important traits for breeding in cassava’). A notable exception is the release of the CR 41–10 variety in Nigeria in 2010 (Fig. 1), which was initially selected using a set of molecular markers linked to CMD resistance, despite the absence of the disease in Colombia [55]. A recent breakthrough identified an SNP within the coding sequence (CDS) of the *phytoene synthase 2* (*PSY2*) gene (Fig. 1), which influences the synthesis of provitamin A carotenoids in cassava’s storage roots [56]. Two SNAP markers were developed to distinguish between homozygous waxy (*wxwx*) and heterozygous nonwaxy cassava genotypes [57]. Diagnostic markers associated with CG accumulation have also been identified in cassava [50]. By conducting molecular marker detection during the seedling stage of the progeny based upon a ‘minimum selection criteria’, it is possible to accurately select germplasm with desirable traits such as high carotenoid content, waxy starch, and low CG content. This targeted approach effectively condenses large breeding populations for subsequent phenotypic recurrent selection cycles, thereby significantly enhancing the overall efficiency of cassava breeding. On the other hand, using GWAS, a significant link was established between 18 allelic variations in heterozygosity within nine genes of interest and seven key agricultural characteristics of cassava. These characteristics include stem height, the number of storage roots per plant, the type of storage root epidermis, storage root weight, the weight of the aerial portion of the plant, resistance to CBB, and starch content [58]. This insight provides a rich array of genetic resources that could be instrumental in the genetic breeding of cassava. MAS has also been effectively utilized to reduce the time required for the introgression of beneficial traits from wild relatives [16]. Together, MAS is highly successful for traits controlled by a single gene, but cannot be used for selection of complex traits in cassava.

### Genomic selection

Unlike traditional MAS, GS assesses the cumulative effect of a broad spectrum of genomic markers. This comprehensive approach leads to the calculation of genomic estimated breeding values (GEBVs), providing a broad-based prediction of an individual’s potential for desirable traits. The precision of GEBV predictions is determined by several critical factors, such as population heterozygosity, the size of the training population, trait heritability, the quality of phenotypic data, and marker density. With at least six high-quality genome sequences available for cassava [33, 59–63], these genomic resources provide a robust foundation for GS in cassava breeding. A notable feature of GS is that it does not require prior identification of QTL within linkage maps, and it can improve multiple traits simultaneously through a selection index, much like traditional phenotypic breeding. One of the foremost advantages of GS is its ability to significantly shorten the breeding cycle. By predicting phenotypic performance from genomic data without the need for full generation growth and observation, GS enables breeders to make selection decisions much earlier. This reduction in cycle time can accelerate the overall breeding process, leading to quicker genetic gains. Additionally, GS allows for the decoupling of population improvement from variety development. Traditional breeding often intertwines these two aspects, as new varieties are developed from the improved traits of a breeding population. With GS, breeders can focus on enhancing the genetic base of a population independently of developing specific varieties. This separation can lead to more efficient breeding methods where population-wide improvements are leveraged to develop multiple varieties tailored to different needs or environments. The potential of GS has been demonstrated in initiatives like the Next Generation Cassava Breeding project, which has documented significant increases in prediction accuracy for diseases like CMD in a Nigerian cassava population [64] and for CBSD severity in Ugandan populations [65]. Research has also shown variability in predictability for yield-related traits and starch properties, underscoring the importance of trait-specific strategies [66]. The complexity of building effective training populations has been highlighted by studies emphasizing the importance of using clones from similar breeding stages for improved prediction accuracy [67].

A challenge for rapid-cycling GS is the tendency to indirectly impose selection pressure on genotypes with high flowering propensity. In turn this favors genotypes with profuse levels of branching and poorer plant architecture. To overcome this undesired consequence, breeders could adopt three strategies: (1) use flower induction methods to ensure balanced crosses are made, including use of erect plant types that are preferred for mechanized planting; (2) include flowering ability and plant architecture in the GS models; and (3) implement GS at stage one testing where parents with good plant type can be selected rather than at seedling nursery. Together, these measures will ensure that GS is effectively used for improving complex traits such as yield and starch content while not compromising aboveground growth characteristics of cassava.

### Genetic transformation

Genetic transformation has proven to be a transformative approach to cassava improvement, effectively addressing the challenges of high heterozygosity and trait segregation associated with traditional breeding methods, and rapidly achieving enhanced target traits. By the 1990s, a robust system for cassava plant regeneration had been established, grounded in somatic embryogenesis, friable embryogenic calli (FEC), and shoot organogenesis from the cotyledons of somatic embryos. Since the first report of transgenic cassava plants by two research teams in ‘*Nature Biotechnology*’ in 1996 [68] (Fig. 1), genetic engineering has steadily advanced over two and a half decades, overcoming genotype constraints and shifting focus from model cultivars to those preferred by farmers [69]. Over the past decade, in addition to the transgenic model variety TMS60444, genetic transformation systems for several cassava cultivars have been established [70–79]. The transgenic recipient tissues are FEC and immature leaf lobe (ILL). For genetic transformation using FECs as the recipient material, the *Agrobacterium* strain LBA4404 is employed, and transgenic seedlings are selected using hygromycin or paromomycin. Among the tested varieties, SC8 demonstrated the highest transformation efficiency. In contrast, for transformation with ILLs as the recipient material, the *Agrobacterium* strain EHA101 is used, and bialaphos is applied for selecting transgenic seedlings, with TMS60444 showing the highest transformation efficiency (Table 1). In these cases, the establishment of the transgenic systems have shed light on discovery of gene functions in cassava. A range of important traits has been targeted for genetic transformation, including improving starch quality and content; increased contents of protein, β-carotene, iron, and zinc; elimination of CG, drought stress, cold stress, and diseases; and pest resistance [80–84], etc. For example, Ruan and colleagues [85] successfully employed *Agrobacterium* LBA4404 carrying the *35S:MeMYB2hpRNA:pCAMBIA1300* construct to transform the FEC of TMS60444, resulting in the development of MeMYB2-RNAi transgenic cassava plants with enhanced drought resistance. Furthermore, Zhou *et al.* [86] developed transgenic lines of TMS60444 with starches rich in up to 50% amylose through the constitutive expression of hairpin double-strand RNA (dsRNA) targeting the 1,4-alpha-glucan-branching enzyme genes. Despite these advancements, the commercial cultivation of transgenic cassava remains elusive, largely due to regulatory hurdles and the challenge of developing varieties that provide clear benefits to producers and processors. For instance, initial attempts at transforming genotypes through somatic embryogenesis aimed to develop varieties resistant to both CMD and CBSD. However, these efforts encountered an unexpected twist: the genetic transformation intended to confer CBSD resistance inadvertently made the plants more susceptible to CMD [87]. Similarly, transgenic cassava and potato plants coexpressing *deoxy-d-xylulose-5-phosphate synthase* (*DXS*) and a bacterial version of *PSY* (*crtB*) exhibited a trade-off between β-carotene accumulation and starch content [88]. Despite these setbacks, transgenic research has yielded valuable insights into gene expression in cassava, paving the way for the transition from developmental to practical applications that bolster cassava industrialization and food security. In light of public concerns over the biosafety of traditional selectable-marker genes, such as those conferring antibiotic resistance and herbicide resistance, there is a pressing need to develop more publicly acceptable marker genes or marker-free technologies in cassava.

**Table 1:** Application progress of cassava genetic transformation.

Genotype	Reporter gene	Selection	Agrobacterium strain	Recipient tissue type	Transgenic efficiency	Reference
TMS60444	gusA	Hygromycin	LBA4404	FEC	10^a^	[70]
TMS60444	gusA	Paramomycin	LBA4404	FEC	14.3–28.6^a^	[71]
T200	gusA	Hygromycin	LBA4404	FEC	5^a^	[72]
TME14	gusA	Paramomycin	LBA4404	FEC	70–80^a^	[73]
KU50	mGFP6	Hygromycin	LBA4404	FEC	33^a^	[74]
SC8	gusA	Hygromycin	LBA4404	FEC	120^a^	[75]
KMM	gusA	Bialaphos	EHA101	ILL	60.15%^b^	[76]
TMS60444	gusA	Bialaphos	EHA101	ILL	75.33%^b^	
08/354	gusA	Bialaphos	EHA101	ILL	0.4%^b^	[77]
KME 1	gusA	Bialaphos	EHA101	ILL	0.4%^b^	
TMS60444	gusA	Bialaphos	EHA101	ILL	0.5%^b^	
BRS222	gusA	Hygromycin	LBA4404	FEC	Unknown	[78]
ADI001	AC1	Hygromycin	LBA4404	FEC	Unknown	[79]

a The rate of obtaining positive transgenic plants per milliliter settled cell volume (SCV).

b The rate of obtaining positive transgenic plants from each ILL explant for genetic transformation.

### Gene editing

A stable gene editing system was established in cassava, which was mainly used to identify gene function but also create some new germplasm. As the reference genome map of cassava continues to be refined, scientists are able to understand and modify the genetic information of cassava with greater precision. The application of CRISPR/Cas9 technology provides an efficient method for the orderly improvement of cassava. The flexibility of the CRISPR/Cas9 technology is reflected in its ability to edit a single specific genomic sequence, known as simple editing, or to target multiple genomic sequences simultaneously, achieving multiplex editing. A CRISPR/Cas9 system was developed to modify *phytoene desaturase* (*MePDS*) in two cassava cultivars (TME60444 and TME204) for the first time [89] (Fig. 1). Consequently, CRISPR/Cas9-mediated modification of *nCBP-1/nCBP-2*, *MeSWEET10a*, *MeEPSPS*, *MeCYP79D1/MeCYP79D2*, *MeGBSS*, *MePTST1*, and *MeSBE2* have resulted in CBSD resistance, CBB resistance, high levels of glyphosate resistance, low CG, and amylose-free in root starch, respectively [90–96]. For instance, Bull *et al.* [93] demonstrated that mutations in two key starch biosynthesis genes, protein targeting to starch 1 (PTST1) and granule-bound starch synthase (GBSS), can reduce or even eliminate amylose content in root starch. Interestingly, the majority of *ptst* lines produced starch with an average amylose content of 13.3%, compared to the 18.5% found in wild-type starch. In contrast, while some *gbss* mutant lines produced amylose-free starch, no *ptst* lines achieved this. This research highlights the immense potential of gene editing in identifying unique lines with varying amylose contents, which is of great interest to the cassava starch industry. Additionally, Hummet *et al.* [94] achieved a significant breakthrough by using CRISPR/Cas9 to modify the 5-enolpyruvylshikimate-3-phosphate synthase (EPSPS) gene, leading to the creation of TME419 plants resistant to high doses of glyphosate, with no detectable T-DNA integrations.

As is shown in Table 2, the current status in cassava gene editing research focusing on editing promoters and coding regions of genes was demonstrated. The editing efficiency for target genes exceeds 87.5%, indicating the high efficiency of the CRISPR/Cas9 system in editing the cassava genome. However, the homozygous mutation rate of cassava gene editing mutants varies significantly across different studies (0%–45%). For example, Odipio *et al.* [89] reported a homozygous editing efficiency of 0% (TME204) or 5% (TMS60444) when using the 35S promoter to drive Cas9 expression for editing the *MePDS* gene. In contrast, Wang *et al.* [75] achieved a homozygous mutation rate of 45% in SC8 by using the same gene editing target but employing the Yao promoter (with high expression in embryos) to drive Cas9 expression. This demonstrates that promoter optimization can further improve the homozygous rate in cassava gene editing. Currently, all cassava gene editing methods involve inserting foreign gene editing components into the genome via T-DNA to achieve the desired gene edits. Bull *et al.* [93] proposed a strategy to eliminate foreign genes through self-crossing. However, due to the highly heterozygous nature of the cassava genome, severe segregation occurs in the progeny, necessitating the reselection of high-quality gene-edited lines from the offspring. This process is labor-intensive and time-consuming. To address this challenge, Mukami *et al.* [97] developed a protoplast regeneration system for cassava leaf cells, laying the groundwork for delivering gene editing tools in the form of ribonucleoproteins (RNPs), enabling cassava gene editing without the introduction of transgenic elements. Although cassava gene editing technology still has some problems such as long cycle and genotype selectivity, it has undoubtedly become an important tool for genetic modification of agronomic traits and gene function validation.

**Table 2:** Application progress of gene editing technology in cassava.

Genotype	Editing system	Target gene	Editing efficiency	Objective	Reference
SC8	CRISPR/Cas9	MePDS	Mutants (93%); homozygous mutants (45%)	Establishing an editing system	[74]
TMS60444	CRISPR/Cas9	MePDS	Mutants (100%); homozygous mutants (5.26%)	Establishing an editing system	[88]
TME204	CRISPR/Cas9	MePDS	Mutants (100%); homozygous mutants (0)	Establishing an editing system	
TMS60444	CRISPR/Cas9	nCBP-1/ nCBP-2	Mutants (87.5% and 95.2% for nCBP-1 and nCBP-2); homozygous mutants (37.5% and 14.3% for nCBP-1 and nCBP-2)	Enhancing CBSD resistance	[89]
TME419	CRISPR/Cas9	MeSWEET10a		Enhancing CBB resistance	[90]
SC8	CRISPR/Cas9	MeSWEET10a	Mutants (98.2%); hmozygous mutants (19.3%)	Enhancing CBB resistance	[91]
TMS60444	CRISPR/Cas9	MeGBSS	Unknown	Creating waxy starch	[92]
TMS60444	CRISPR/Cas9	MePTST1	Unknown	Creating waxy starch	
TME419	CRISPR/Cas9	MeEPSPS	Unknown	Enhancing glyphosate resistance	[93]
TME419	CRISPR/Cas9	MeCYP79D1/ MeCYP79D2	Unknown	Reducing CG content	[94]
TMS60444	CRISPR/Cas9	MeCYP79D1/ MeCYP79D2	Unknown	Reducing CG content	
TMS91/02324	CRISPR/Cas9	MeCYP79D1/ MeCYP79D2	Unknown	Reducing CG content	
TMS60444	CRISPR/Cas9	MeSBE2	Mutants (93.02%)	Increasing amylose content	[95]

## Continuous updates and optimization of cassava reference genome

**Figure 2:**
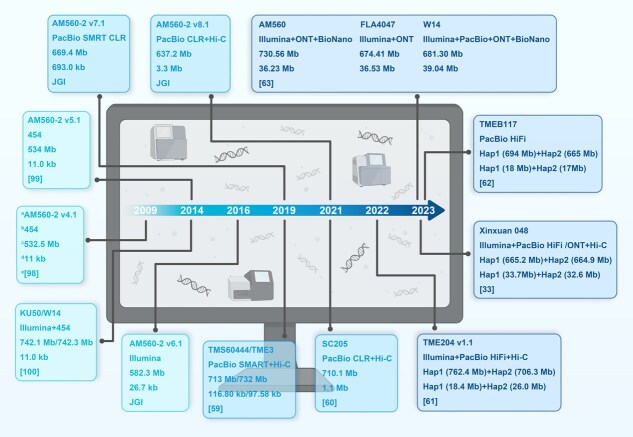
Timeline of cassava reference genome sequencing data. The superscript letter a, b, c, d, and e represent the genotype/version, primary sequence technology, genome size, contig N50 length, and the cited reference, respectively.

Genomic sequence data are paramount for advancing our understanding of gene evolution, genomic variation, gene regulation, and plant breeding. In present, at least six reference genomes with near-T2T sequences as well as a pan-genome have been released in cassava [33, 59–63]. The evolution of genome sequencing technologies and bioinformatic methodologies in the context of cassava is illustrated in Fig. 2 for reference.

The first draft genome, version 4.1 (v4.1), was assembled using 454-based sequencing of the inbred line AM560–2 (S2 of AM560) (Fig. 1), resulting in a total scaffold length of 532.5 Mb in 2009 [98]. The v5.1 improved upon v4.1 by reorganizing it into a chromosome-scale format, which integrated and oriented 57% of the sequences from v4.1 into 18 chromosomes [99]. The genomes of W14 (*M. flabellifolia*) and KU50 were assembled, using a combination of Illumina Hiseq2000 and 454 sequencing platforms [100]. The v6.1 assembly marked a notable leap forward from its predecessor, v5.1, by introducing an Illumina-based approach for AM560–2. This new version expanded the total contig sequence by 18%, significantly improving the continuity of the sequence and integrating an additional 45% of the sequence into chromosomal scaffolds, as reported by the Joint Genome Institute (JGI). Building on the foundation laid by v6.1, v7.1 adopted a similar strategy but took it a step further by incorporating more contiguous sequences generated *de novo* from PacBio single-molecule real-time (SMRT) continuous long-read (CLR) data. This comprehensive assembly spans 669.4 Mb and encompasses 33 849 protein-coding genes. Although slightly shorter than the anticipated 750 Mb for the haploid genome size, it boasts a contig N50 of 693 kb, demonstrating a substantial improvement in sequence assembly quality (JGI). This progress underscores the ongoing advancements in genomic sequencing technologies and their application in enhancing our understanding of the cassava genome. The v8.1 assembly integrates High-throughput Chromatin Conformation Capture (Hi-C) data and long-range linking information from the v7.1 assembly, with Hi-C technology anchoring an additional 187.8 and 26.0 Mb of sequence over the v6.1 and v7.1 assemblies, respectively. This enhancement resulted in a contig N50 of 3.3 Mb (JGI). Utilizing a combination of PacBio SMAR and Hi-C technology, Kuon *et al.* [59] have reported the assemblies of TMS60444 and TME3, providing phased haplotype information for >80% of the genomes. The deduplicated primary haploid assembly reached a total size of 732 Mb for TME3 and 713 Mb for TMS60444. In 2019, remarkable progress was made in the genome sequencing of the cassava plant, with the assembly of an allele-defined genome for SC205. This process led to the identification of 24 128 biallelic sites across 18 pairs of homologous chromosomes, encompassing 63.6% of the total annotated genes [60]. Meanwhile, the haplotye-resolved chromosome pairs of the African cassava cultivar TME204 were assembled (v1.1) using a combination of PacBio CLR/High-fidelity (HiFi) and Hi-C technology, providing insights into allele-specific expression [61]. Update of HiFi technique of PacBio and ultralong sequencing technology of Nanopore, as well as emergence of novel assembly algorithms, enabled a no-gap reference genome, the so-called T2T genomes [101, 102]. The first cassava T2T genome of Xinxuan 048 achieved a remarkable assembly with no gaps in 11 chromosomes, utilizing a combination of PacBio HiFi, Oxford Nanopore Technologies (ONT), and Hi-C (Fig. 1). Within Xinxuan 048 haplotype-resolved genomes, a total of 929 Mb of transposable elements (TEs) were identified, constituting 69.86% of the genome [33]. A similar work also has been reported with TMEB117 [62]. The near-T2T genomes of AM560, wild ancestor FLA4047 and W14, as well as a pan-genome comprising 30 representative wild and cultivated accessions of cassava has been released. Comparison analysis of SVs and SNVs between the ancestors and cultivated cassavas has revealed significant expansion and contractions of genes and gene families. Notably, selective sweeps have occurred in 122 genimic regions, implicating 1519 candidate domestication genes. The evolution of photosynthesis, flowering, and storage root initial and CG was unlocked [63]. Collectively, the genomic resources of cassava are indispensable for subsequent reference-guided omics analyses and for elucidating the genetic underpinnings of key agronomic traits.

## Gene mining of important traits for breeding in cassava

**Figure 3:**
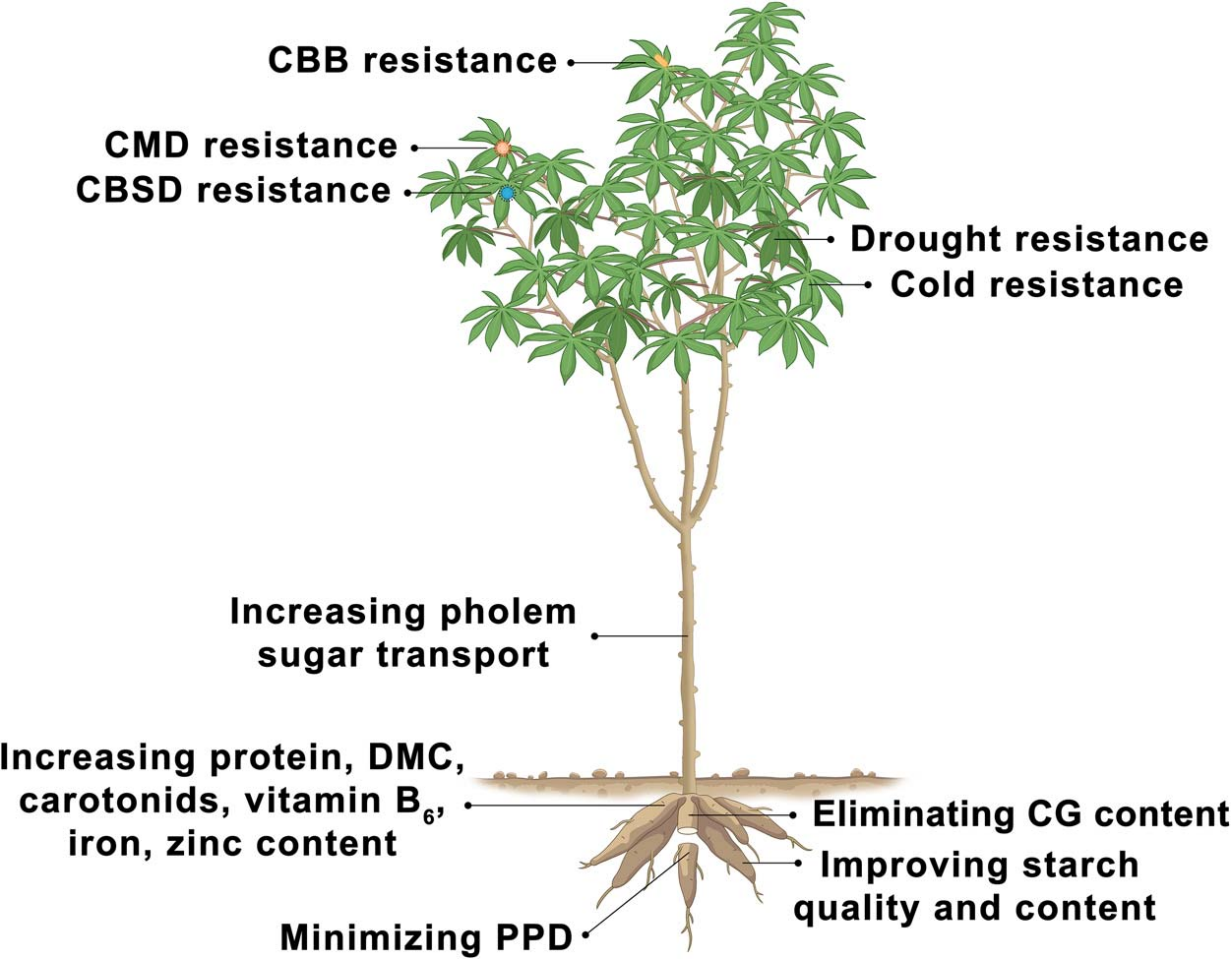
Plant architecture and decomposition of the important traits in cassava

In this section, we summarize the up-to date functional genes controlling important traits, including starch quality and content, dry matter content (DMC), tolerance to PPD, nutritional quality (protein levels, carotenoids, vitamin B_6_, iron, zinc content, and CG concentrations), drought and/or cold resistance, and disease stress (CMD, CBSD, and CBB) (Fig. 3; Supplementary Table S1).

### Starch quality and content

The industrial sector is witnessing a persistent and escalating demand for cassava varieties that not only exhibit superior starch quality but also feature novel types of starch and an enhanced starch content. The storage roots of cassava are remarkable for their ability to achieve a starch content that typically is as high as 32% of its fresh weight. Starch is a complex carbohydrate composed of two primary components: amylose and amylopectin. Amylose is a linear polymer characterized by α-1,4-glycosidic linkages, and it constitutes ~20%–30% of the overall starch content. In contrast, amylopectin is a highly branched molecule linked by α-1,6-glucosidic bonds, making up 70%–80% of most cassava starches [96]. The presence of amylose significantly impacts the physicochemical properties of starch during the cooking and processing phases. The viscosity and gelatinization characteristics of starch from amylose-free cassava varieties are more suitable for various industrial applications, especially in applications requiring high transparency, good solubility, and high viscosity stability. The valuable trait of amylose-free starch has been successfully introduced into cassava through innovative approaches, either by employing transgenic RNA-based techniques [103] or by screening self-pollinated lines to identify natural recessive mutants [40].

Starch synthesis occurs within the plastids, specifically in chloroplasts and amyloplasts found in leaves and storage tissues, respectively. This process is facilitated by three primary pathways: the Calvin cycle, sucrose synthesis, and the biosynthesis of storage starch. The carbon source for starch biosynthesis in storage organs is predominantly sucrose, which is transported from the leaves to the storage roots. The mechanisms of sucrose unloading, encompassing both symplastic and apoplastic pathways, are pivotal in the transport of photoassimilates, with the choice of pathway being species-, organ-, and tissue-specific [104, 105]. In general, the apoplastic pathway is predominant in the transport of sucrose and other assimilates in the fibrous roots of tuberous crops [106]. However, during the initial stages of tuberization and storage root formation, the mode of unloading shifts to the symplastic pathway. This transition is accompanied by an increase in the activity of sucrose synthase (SuSy), an enzyme that catalyzes the conversion of sucrose and a nucleoside diphosphate into nucleoside diphosphate glucose and fructose, and a decrease in the activity of cell wall invertase (CWINV), which is crucial for sugar hydrolysis during phloem unloading in the storage organs of sweet potato (*Ipomoea batatas*) and cassava [107, 108]. Overexpression of *SuSy* in potato tubers significantly increased the content of starch, UDPglucose, and ADPglucose [109]. Conversely, overexpression of *MeCWINV3* in cassava inhibited sugar export to storage roots and accelerate sucrose hydrolysis in leaves, resulting in a decrease in storage root yield [110].

The biosynthesis of starch is a complex process that requires the coordinated action of multiple enzymes involved in starch synthesis. ADP-glucose pyrophosphorylase (AGPase) is instrumental in catalyzing the conversion of glucose-1-phosphate (Glc1-P) and ATP into the activated glucosyl donor, ADP glucose. AGPase is considered as an important rate-limiting enzyme in starch biosynthesis pathway in cassava storage roots. GBSS is primarily associated with amylose production and is encoded by a highly conserved gene that is uniquely present in cassava [57]. The synthesis of amylopectin is a collaborative effort involving soluble starch synthase (SSs), starch branching enzyme (SBE), and starch debranching enzymes (Isoamylase, ISA; Pullulanase, PUL) [52]. Silencing the gene encoding the large subunit of AGPase, *MeAPL3*, in transgenic cassava lines resulted in a significant reduction in storage root starch [111]. High-amylose storage roots can be engineered in cassava by targeting the function of *MeSBE1* and *MeSBE2* [86, 96]. In addition, in *Arabidopsis* chloroplasts, PTST1, which carries an N-terminal coiled-coil domain and a C-terminal carbohydrate-binding module, mediated GBSS localization on starch granules. Targeted mutagenesis of *GBSS* or MePTST1 produced low-amylose cassava storage root [93].

In conclusion, *MeAPL3*, *MeGBSSI*, *MePTST1*, *MeSBE1*, *MeSBE2*, and *MeCWINV3* are identified as potential target genes for the enhancement of starch content in cassava storage roots, offering promising avenues for future research and development in the field.

### Dry matter content

DMC is a critical component of cassava’s dry yield, fundamentally influencing the overall performance of the crop and its acceptance by growers, processors, and consumers alike. DMC in cassava storage roots can vary significantly, with a range from 17% to 47%. The majority of varieties display DMC levels between 20% and 40%, with those >30% being considered as having high DMC [112]. Importantly, DMC has been found to have a robust positive correlation with starch content and HI, and a negative correlation with provitamin A carotenoids [113]. This highlights the importance of these traits in effective indirect selection strategies. Significant advancements have been made in understanding the genetic basis of DMC in cassava, with two major QTLs identified on chromosomes 1 and 6 [48]. The most prominent of these loci encompasses two crucial genes involved in starch and sucrose metabolism: Manes.01G123000, encoding UDP-glucose pyrophosphorylase, and Manes.01G123800, encoding sucrose synthase. These genes are in close genomic proximity to *PSY2* (Manes.01G124200) (v6.1 genome), aligning with findings from Rabbi *et al.* [113]. Yet, the specific mechanisms—whether physical linkage or pleiotropy—responsible for the negative correlation between DMC and provitamin A carotenoids in cassava remain unclear. Potential follow-up experiments could involve exploring the impact of gene editing technologies, such as CRISPR/Cas9, on yellow-rooted genotypes by knocking out *PSY2* to determine if this results in an increase in DMC. Additionally, as previously mentioned, knockout of *MeAPL3* significantly reduced both storage root starch and DMC [111]. These genes represent valuable targets for further genetic research and may hold the key to understanding the genetic factors that contribute to DMC variation in cassava.

### Postharvest physiological deterioration

**Figure 4:**
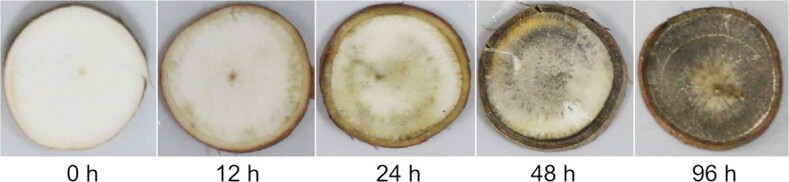
Cassava roots showing progression of PPD from 0 to 96 h after harvest

Globally, PPD rate of cassava storage roots are estimated to be ~19% [114]. PPD manifests in cassava storage roots as a discoloration to black-blue or brown, primarily affecting the cross-section, followed by the emergence of dark streaks on the xylem’s vascular vessels (Fig. 4). To our knowledge, no precise QTL associated with PPD have been identified thus far except the large confidence interval utilizing the single-marker regression approach reported by Fernando *et al.* [115]. Although its specific regulatory mechanism is still unclear, great progress has been made in recent years. The phenomenon of PPD is intricately linked to various biochemical processes, including the generation of reactive oxygen species (ROS), calcium signal transduction pathways, starch degradation, CG biosynthesis, phenylpropanoids biosynthesis, and N-glycosylation modifications [114, 116, 117]. Regulating gene expression in the storage root is a promising strategy to delay PPD. Recently, Mbinda and Mukami [118] have summarized many crucial genes conferring PPD tolerance in a review article. For instance, virus-induced gene silencing (VIGS) of *chalcone synthase 3* (*MeCHS3*) and *anthocyanidin reductase* (*MeANR*) has revealed changes in tolerance to PPD and alterations in the total flavonoid and anthocyanin contents in leaves [119]. Silencing of *MeAPL3* resulted in plants displaying significant reduction in storage root starch and DMC and reduced or no PPD compared to controls, suggesting a direct relationships between starch/dry matter and its role in PPD [111]. An early surge in ROS is observed during PPD [120]. The silencing of *peroxidase 12* (*MePOD12*) results in tuberous roots that decay more rapidly than wild-type cassava, with reduced antioxidant capacity and lignin content [121]. The coordinated expression of cassava *copper/zinc superoxide dismutase* (*MeCu/ZnSOD*) and *catalase 1* (*MeCAT1*) enhanced the synergistic ROS-scavenging ability of cassava storage roots postharvest, thereby delaying the onset of PPD [122]. Melatonin has emerged as a promising candidate for mitigating PPD in cassava, owing to its potent ROS-scavenging capabilities [123]. Recent findings suggest that CGs are known to shorten the shelf life of harvested cassava storage roots by inhibiting mitochondrial respiration through cyanide release, leading to increased ROS production that exacerbates PPD [124]. Transgenic cassava plants overexpressing a codon-optimized *Arabidopsis* mitochondrial alternative oxidase gene (*AOX1A*), which is insensitive to cyanide, have demonstrated an extended storage root shelf life of up to 21 days under both greenhouse and field conditions by reducing ROS accumulation [125]. Furthermore, Yan and colleagues have shown that the application of exogenous abscisic (ABA) and ethephon can mitigate the symptoms of PPD in sliced cassava tuberous roots [126, 127]. ABA mitigates PPD symptoms by activating the MePYL6 − MePP2C16 − MeSnRK2.1 − MebZIP5/34 − MeGRX6/ MeMDAR1 signaling module, which in turn reduces the levels of endogenous hydrogen peroxide. The genes identified within this module are promising candidates for enhancing the postharvest longevity of cassava [126]. Ethephon, on the other hand, enhances the antioxidant defense by diminishing the levels of malondialdehyde and H_2_O_2_, and by modulating energy metabolism pathways, which results in a delay of PPD [127]. Taken together, these findings demonstrate the great potential of biotechnological tools in mitigating the effects of PPD.

From the point of view of plant reproduction, the cultivated cassava produces its offspring from the stalk, and the storage root is the storage organ that provides nutrients for the stalk. It is different from potato and sweet potato, which have a dormancy process for the reproductive organs, while the former does not have a dormancy habit. Understanding these unknown differences can provide the key to finally unlock the mechanism of PPD in cassava.

### Nutritional quality

Micronutrient deficiencies, often referred to as ‘hidden hunger’, impact over half of the global population, with a particularly detrimental effect on the health of women and young children in developing nations [128]. Cassava storage roots accumulate starch and sugar efficiently, but lack protein and other nutrients. Therefore, with the goal of food use, nutritional fortification toward increasing nutritional quality has been widely paid attention to and achieved remarkable results. Several biotechnological events enhanced protein, carotenoids, vitamin B_6_, iron, and zinc contents in cassava [129, 130].

### Protein content

Cassava storage roots are known for having some of the lowest protein content among major food crops, typically ranging from 0.7% to 3% by DW [131]. To combat protein energy malnutrition associated with a diet heavily reliant on cassava, efforts have been made to increase the protein content in cassava roots through advanced breeding techniques and genetic modification. In one significant approach, the *aspartic proteinase 1* (*ASP1*) gene, which encodes an artificial storage protein, was introduced into transgenic cassava storage roots under the control of the CaMV 35S promoter [132]. Additionally, a novel recombinant storage protein named zeolin, engineered from corn gammazein and bean phaseolin, was successfully expressed specifically in the storage roots under the control of the root-specific patatin promoter. Furthermore, overexpression of *hydroxynitrile lyase* (*HNL*) has been shown to not only increase protein content but also to reduce residual CG levels in cassava roots [133]. This suggests that the improvement in CG metabolism plays a role in elevating root protein levels in cassava. Such efforts can contribute to alleviating malnutrition and improving the overall food security and health of populations that rely heavily on cassava as a staple food.

### Carotenoids content

Vitamin A deficiency, typically stemming from a lack of plant-derived carotenoids in the diet, is a leading cause of illness, blindness, and mortality from infectious diseases among children [134]. In nature, yellow-rooted cassava cultivars producing provitamin A carotenoids are rare; most breeding populations are white-rooted. By inserting *crtB,* the bacterial version of the plant *PSY2*, in the white roots of the TMS60444 cassava variety, the total carotenoid content was increased to 22 and 7 μg/g DW of β-carotene [56, 135]. Further transgenic work, combining the bacterial genes *crtB* and *DXS*, has pushed the total carotenoid content even higher, reaching a maximum of 60 μg/g DW [136]. Dissection of genetic architecture of pro-vitamin A previously identified genomic regions on chromosome 1 around *PSY2* [137]. Therefore, *PSY2* has great application prospects in improving the accumulation of vitamin A in cassava roots. More recently, five new SNPs associated with this trait on chromosomes 5, 8, 15, and 16 [48]. These SNPs provide a practical foundation for enhancing the carotenoid content in cassava roots through MAS and GS, paving the way for the cultivation of cassava varieties with enriched nutritional profiles to combat vitamin A deficiency.

### Vitamin B_6_ content

Vitamin B_6_, also known as pyridoxine, is crucial for human health, playing a vital role in preventing a spectrum of conditions, including cardiovascular disease, diabetes, and neurological disorders. Since humans are incapable of synthesizing vitamin B_6_  *de novo*, this essential micronutrient must be obtained through dietary intake and intestinal absorption. Analysis of micronutrient content in raw cassava roots suggests that a moderate increase of 2.3 times the current vitamin B_6_ content could suffice to meet the recommended dietary allowance for populations with a heavy reliance on cassava in their diet [138]. Two pivotal enzymes for vitamin B_6_ biosynthesis in plants are PDX1 (a synthase) and PDX2 (a glutaminase) [139]. Additionally, heterologous expression of two *Arabidopsis* genes, *AtPDX1.1* and *AtPDX2*, in cassava led to a 4- to 48-fold increase in total vitamin B_6_ content in leaves and a 2- to 6-fold increase in roots [140]. These transgenic plants have the potential to provide the required daily intake of 1.3 mg of vitamin B_6_ for an adult by consuming as little as 10 times the amount of boiled storage roots.

### Iron and zinc content

In Nigeria, a significant health concern is prevalent among children, with 39% of those aged 0–19 suffering from anemia primarily attributed to iron deficiency, and a staggering 63% of children aged 0–5 experiencing zinc deficiency [141]. These micronutrient deficiencies can have profound effects on children’s health, including the impairment of immune function, stunted growth, and hindered cognitive development [142]. To address this issue, a notable example of biofortification is overexpression of the iron assimilatory gene *FEA1* in cassava roots. This targeted genetic enhancement has successfully elevated iron levels in the roots, increasing from 10 to nearly 40 ppm [143]. Another example is the introduction of the vacuolar iron transporter gene, *AtVIT1* in TME204. This genetic modification resulted in a 3- to 4-fold increase in iron concentration within the storage roots [144]. In a similar vein, cassava lines engineered to coexpress a mutated iron transporter gene *AtIRT1*, along with the ferritin gene, *AtFER1*, have exhibited remarkable iron accumulation levels that are 7–18 times higher and zinc levels 3- to 10-fold higher than conventional cultivars under field conditions [141]. Recently, a significant milestone was achieved by developing a cassava cultivar harboring the *AtIRT1* and *AtFER1* transgenes. This breakthrough has led to the production of cassava storage roots with nutritionally significant levels of iron and zinc, at 145 and 40 μg/g DW, respectively [145].

### Cyanogenic glucoside content

CG is a widespread class of compounds found across various plant species, including *Sorghum bicolor*, *M. esculenta*, *Hevea brasiliensis, Prunus amygdalus*, and *Lotus japonicus*, etc [146]. These compounds can release toxic hydrogen cyanide (HCN) upon the hydrolysis of their β-glycosidic bond by β-glycosidase enzymes. Sweet cassava is characterized by their low CG content—<50 mg/kg of fresh root weight—and is considered ‘bitter’ when this threshold is exceeded. CG content in cassava storage root was characterized as a quantitative trait. Five QTLs affecting CG were identified across four linkage groups (LG), namely LG 2, 5, 10, and 11 [147]. Notably, on LG 10 and 23, two QTLs exhibited additive effect on CG content in cassava storage root [148]. Most encouragingly, two major loci were identified: one is a gene that encodes a plasma membrane H^+^-ATPase (Manes.14G073900), and the other is a gene that encodes a multidrug and toxic compound extrusion (MATE) protein (Manes.16G007900). The two genes are substantial contributors, accounting for up to 7% and 30% of the HCN concentration in the roots, respectively [50].

Cassava plant contains two primary CGs: linamarin and lotaustralin, with linamarin being the predominant form, accounting for ~95% of the total CGs [149]. Various factors influencing the distribution of CG, including source–sink relationships, transport controls, soil nitrogen availability, and environmental conditions, have been clarified, but the specific regulatory factors that determine the CG content in cassava leaves or roots remain unclear [124]. The initial and rate-limiting step in the biosynthesis of CG is facilitated by cytochrome P450 enzymes, CYP79D1 and CYP79D2, which convert valine into a valine-oxime [150]. This is followed by the transformation of oximes into cyanohydrins by CYP71E7 [151]. The final step in this process is the glycosylation of cyanohydrins, which is carried out by UDP-glucosyltransferase, UGT85K4/K5 [152]. Interestingly, *CYP79D2*, *UGT85K4*, *CYP71E7*, and *UGT85K5* are structurally clustered on chromosome 12, which may enhance their coordinated expression and the likelihood of inheriting beneficial genetic variations together [153]. This knowledge has been instrumental in developing molecular strategies aimed at reducing the linamarin content in cassava roots by manipulating the sink–source relationships. CGs are primarily biosynthesized in the shoot apex and transported to the storage roots via the phloem [154]. Genetic studies have shown that the knockout of *CYP79D1* and *CYP79D2* results in the absence of CGs in both the leaves and storage roots of cassava [96]. This knowledge has been instrumental in developing molecular strategies aimed at reducing the linamarin content in cassava roots by manipulating sink–source relationships. In addition, overexpression of *HNL* in cassava roots led to substantially reduced CG levels and increased total protein content [133]. This suggests a potential competition between linamarin storage and amino acid synthesis in the vacuole, possibly due to an enhanced sink strength for the assimilation of CGs into proteins.

In summary, CG is crucial in the nitrogen metabolism of cassava and should not be viewed merely as byproducts of cassava plant metabolism. The associated risk of cyanide toxicity makes the manipulation of CG metabolism and the reduction of root cyanide toxicity a complex task. However, by strategically directing cyanogens away from storage and toward protein synthesis, it may be feasible to ultimately decrease the toxicity of cassava root.

### Drought and/or cold stress resistance

As climate change progresses, it ushers in an era characterized by increased temperatures and arid conditions. Over the past decade, the global impact of drought on crop production has been staggering, with losses amounting to ~$30 billion [155]. The drought stress responses in cassava encompass early stomatal closure; a reduction in photosynthetic protein levels; reduction of growth; an increase in proline and ABA concentrations; enhanced shoot height, root number, and photosynthetic pigments; elevated endogenous CAT and SOD activities; a deep root system; and the shedding of older leaves, among others [156, 157]. Additionally, water stress can not only decrease starch yield in cassava but also modify the functional attributes of starch granules [158]. Higher content of CG in the young leaves and tubers of the cassava variety Mcol 1468 have been observed under water-deficit conditions [159]. Recent studies have identified a range of genes including transcription factors, noncoding RNAs, and enzymes, which connected the complex and flexible transcriptional regulatory network involved in drought and/or cold stress responses in cassava. ROS scavenging and plant hormone signaling pathways are closely associated with abiotic stress signaling pathways, and large amounts of data on their crosstalk are available [81, 83, 85, 160–173]. For example, a CC-type glutaredoxin (GRX) known as MeGRXC3 was identified as a negative regulator of drought resistance. It modulated stomatal closure induced by drought and ABA, playing an antagonistic role in the accumulation of hydrogen peroxide (H_2_O_2_) within epidermal cells and guard cells. MeGRXC3’s influence extended beyond its interaction with two catalases; it also engaged with a TGA transcription factor (MeTGA2), thereby regulating the expression of *MeCAT7* and influencing the homeostasis of ROS [81]. Additionally, the expression of a *SQUAMOSA promoter binding protein-like 9* (*MeSPL9*) was significantly repressed under drought treatment. Overexpression of a dominant-negative form of miR156-resistant *MeSPL9* conferred drought resistance in cassava. The transgenic cassava lines demonstrated not only an increase in osmoprotectant metabolites including anthocyanin and proline, but also accumulated higher levels of endogenous jasmonic acid (JA) and soluble sugars [169]. Transgenic cassava plants engineered to coexpress the *MeCu/ZnSOD* and *ascorbate peroxidase 2* (*MeAPX2*) have demonstrated a notable increase in resistance to both oxidative and chilling stresses [163]. By enhancing the activity of these key antioxidant enzymes, the transgenic cassava exhibits improved resilience, making it a promising avenue for developing crops that can better withstand the challenges of climate change.

Together, we outline the current knowledge of drought and/or cold stress-related genes and their regulatory networks in cassava. However, it remains unknown how cassava plants sense and transmit drought and/or cold stress signals to regulate gene expression. In the future, cassava genome resequencing and gene editing technology for generating mutants are anticipated to uncover further insights into the signaling components. This advancement will provide a more comprehensive understanding of the intricate signaling networks involved in response to drought and potentially cold stress. This progress is set to provide a more comprehensive understanding of the intricate signaling networks that govern responses to drought and/or cold stress, thereby paving the way for the development of cassava varieties with enhanced resilience to environmental challenges.

### Disease resistance

Cassava production is notably impeded by two viral diseases—CMD and CBSD—and the bacterial disease, CBB. The African cassava mosaic virus (CMV), responsible for CMD, and *Xanthomonas axonopodis* pv. *manihotis* (*Xam*), the pathogen causing CBB, are recognized as the world’s sixth and seventh most critical viral and bacterial pathogens, respectively, featuring on the top 10 list of globally significant pathogens [174]. CBSD has had a profound economic impact since its emergence in Eastern Africa, with annual yield and financial losses in Tanzania alone estimated at $51 million [175, 176]. The cultivation of disease-resistant varieties is a strategic and forward-thinking approach to bolster cassava productivity. Over the past two decades, genetic engineering has attracted considerable interest for its potential to produce virus-resistant cassava. This has been notably achieved through RNA interference (RNAi)-mediated resistance, which primarily involves post-transcriptional silencing of viral genes [177, 178]. The genetics and advancements in genomics also have illuminated new pathways for the discovery of natural resistance sources, offering a beacon of hope for the development of resilient cassava varieties.

### Cassava mosaic disease resistance

Three distinct forms of CMD resistance have been identified. The first, known as *CMD1*, is characterized as a recessive polygenic trait that has been mapped using SSR markers, initially introduced from the wild cassava relative *M. glaziovii* in Tanzania [179]. The second form, *CMD2*, stands out as a significant monogenic, dominant trait that has been identified in African landraces [180]. *CMD2* confers resistance to all known CMVs [181]. Extensive studies utilizing both SSR markers for biparental QTL analyses and SNP-based GWAS have been undertaken to unravel the genetic underpinnings of qualitative resistance to CMD. While there are indications from some research that suggest the presence of additional resistance loci, the preponderance of evidence converges on the *CMD2* locus as the primary determinant [180–183]. The first study identified two markers, an SSR (SSRY28) and an RFLP (GY1) that flank a single locus named *CMD2* at distances of 9 and 8 cM, respectively [180] (Fig. 1). Rabbi *et al.* [181] mapped CMD resistance locus that occurs in the vicinity of an SNP marker S5214_780931, which is just 623.24 kb away from SSRY28 (between 157 470 and 157 616 bp, v6.1 genome). Kuon *et al.* [59] assembled long haplotypes of the *CMD2* locus in both TMS60444 (CMV-susceptible) and TME3 (CMV-resistant) to narrow the large *CMD2* region. Two *POD* genes, Manes.12G076200 and Manes.12G076300 (v6.1 genome), were identified to the candidate *CMD2* [59], which supported the GWAS results of Wolfe *et al.* [182]. However, the sustainability of this resistance has been a subject of concern, primarily due to its confined geographical origin and the swift evolutionary pace of cassava mosaic geminiviruses. The apprehensions are heightened by the observed loss of *CMD2* resistance in plants that have undergone somatic embryogenesis in tissue culture [73]. Encouragingly, Lim *et al.* [184] have elucidated that both the *CMD2* resistance and its loss of *CMD2* resistance phenotypes have a genomic basis, colocalizing on the cassava genome. This breakthrough was achieved through whole-genome sequencing and genetic variant analysis, revealing a nonsynonymous SNP in the *DNA polymerase δ subunit 1* (*MePOLD1*) that cosegregates with the *CMD2* resistance within a refined locus of 190 kb. The mutations in *MePOLD1* are postulated to introduce replication errors in geminiviruses, potentially impairing their replication efficiency and reducing the viral load in the host plant. To cope with CMD, cassava breeders are advised to explore alternative sources of CMD resistance. This may include the use of cytokinin meta-topolin [6-(3-hydroxybenzylamino) purine] to stimulate *in vitro* shoots on nonembryogenic explants [185]. The presence of satellite viruses, identified as exacerbating agents in the symptomatology of geminiviruses, complicates the situation by potentially undermining the *CMD2* resistance [186]. Given these complexities, there is an urgent need to regularly reassess the mechanisms of CMD resistance to ensure their continued efficacy against the evolving challenges posed by the geminiviruses. Innovative methodologies, such as gene pyramiding and broadening the genetic base of resistance sources, are gaining recognition as auspicious tactics to fortify cassava’s disease resilience. The utilization of genomic resources, including SSR and SNP genotyping through next-generation sequencing, combined with avant-garde biotechnologies like gene editing and genetic engineering, can alter the susceptibility of certain genes and bestow newfound resistance. These cutting-edge strategies have the potential to markedly transform the landscape of cassava cultivation. The third domain of CMD resistance, designated as *CMD3*, is characterized by two additive regions that complement *CMD2*. These regions are hypothesized to interact in an epistatic manner, implying that their combined effect is greater than the sum of their individual contributions to the resistance phenotype [182]. To forge a long-lasting defense, the amalgamation of monogenic and polygenic resistance sources is advocated as a formidable enhancement strategy. Confronted by the mutable nature of the virus, the quest for additional resistance sources or the implementation of biotechnological advancements like gene editing may offer efficacious and sustainable solutions.

### Cassava brown streak disease resistance

In contrast to CMD, for which resistance breeding is well established and effectively implemented, the development and application of strategies for CBSD tolerance have not been as thoroughly pursued. This is primarily attributed to the relatively low levels of resistance observed, the absence of a comprehensive characterization of CBSD in resistant genotypes, and the complex interactions between genotype and environment. The origins of resistance to CBSD can be traced back to the species *M. glaziovii* and *Manihot melanobasis*, with breeding efforts initiated in Tanzania during the late 1950s and early 1960s. Following a series of greenhouse experiments and extensive field trials, three genotypes—COL 40, PER556, and COL 2182—have been identified as being immune to CBSD [187]. Unlike CMD, resistance to CBSD is predominantly polygenic. Notably, two consistent QTLs on chromosomal 2 and 12 across seasons were detected for resistance to CBSD-induced root necrosis and foliar symptoms in the cultivar Namikonga. Within 253 kb on chromosomal 11 (5507842-5 761 172 bp, v6.1 genome), 27 genes were identified including two leucine-rich repeat (LRR) proteins and a signal recognition particle, which are believed to contribute to CBSD resistance [183]. Further mapping efforts have successfully pinpointed two QTLs associated with CBSD resistance. One is located on chromosome 4, aligning with an introgression segment from *M. glaziovii*, and the other on chromosome 11, which encompasses a cluster of nucleotide-binding site-leucine-rich repeat (NBS-LRR) genes [65]. These findings underscore the genetic complexity and potential pathways for developing CBSD-resistant cassava varieties. Gomez *et al.* [90] leveraged the CRISPR/Cas9 gene editing technology to target cassava eIF4E isoforms, specifically the novel cap-binding proteins 1 and 2 (nCBP-1 and nCBP-2). This groundbreaking approach yielded mutants with delayed and attenuated CBSD aerial symptoms and a reduction in the severity and incidence of storage root necrosis. These advancements highlight the potential of modern biotechnological tools in bolstering cassava’s disease resistance and securing the crop against devastating diseases.

### Cassava bacterial blight resistance

Similar to CBSD, resistance to CBB is polygenic, with regions of resistance located near *CMD2* [180]. Mapping of resistance sources in 150 intraspecific crosslines of cassava revealed eight QTLs that could aid in breeding efforts [54]. However, the resistance level associated with these eight QTLs was found to vary seasonally. Recent researches have pinpointed many CBB resistance genes (including the sensitive allelic genes) in cassava through reverse genetics approach, as listed in supplementary Data Table 1 [91, 92, 188–194]. For instance, four NBS-LRR genes were shown to positively modulate CBB resistance against *Xam* by regulated endogenous salicylic acid (SA) and ROS accumulation, as well as the transcription of pathogenesis-related gene 1 (PR1) [179]. SA serves as a secondary messenger for systemic acquired resistance, and its production is indicative of a plant’s successful recognition of pathogen infection and activation of pathogen-associated molecular pattern (PAMP)-triggered immunity (PTI) and effector-triggered immunity (ETI) [195]. Growing evidences indicate that SA also plays a pivotal role in regulating CBB resistance in cassava [178–180]. The transcription factor MeHsf3 was implicated in the regulation of CBB resistance through modulation of SA accumulation [188]. Moreover, MeHSP90.9 interacted with SHI-related sequence 1 (MeSRS1) and MeWRKY20 to activate SA biosynthesis and accumulation, thereby enhancing resistance to CBB [191]. Consequently, the levels of endogenous SA accumulation are considered a biomarker for CBB resistance in cassava.

Collectively, more CBB resistance genes would be cloned in order to gain a better picture of signaling networks. Moreover, the application of forward genetics techniques may offer new avenues for enhancing the CBB resistance, thereby fortifying cassava against one of its most pernicious diseases.

## Conclusion and perspective

In this review, we have outlined the current status of cassava germplasm resources globally, the evolution of cassava breeding methods from conventional to genomic approaches, the advancements in cassava genome assemblies, and the diverse studies undertaken to enhance key traits. Although promising progress has been made in cassava genetic improvement, the potential for developing functional varieties from the perspective of genetic resource utilization is far from being realized. To resolve the problem of high heterozygosity of cassava genome, isolated inbreeding could be used to create near-pure lines of more than four generations of self-cross. Based on the inbreeding population with large individual lines,it is feasible to establish a practical GS breeding model with genome resequencing combined with accurate phenotype identification. Extremely, super-hybrids could be bred by selectively crossing between pure lines with different geographical backgrounds. However, we need to overcome the multigeneration inbreeding depression and the challenge of the large workload of multiple parent inbreeding through selection. Another transformative avenue in cassava research is the development of a protocol for the production of doubled haploids (DH). The generation of DH through anther or microspore culture could significantly streamline the process of obtaining homozygous cassava genotypes. This innovation could substantially reduce the time required to develop genetically uniform lines, thereby accelerating the breeding cycle and enhancing the genetic improvement of cassava.

Hybridization and genome duplication are fundamental driving forces of evolution in plants, whether they occur naturally or are induced through human intervention [196, 197]. They can change the composition of the genome, increase genetic diversity, and change gene dosage and function, fueling trait innovations [198–200]. Evidence, albeit limited, has shown that artificially generated triploid plants—such as the triploid groups of Populus (*Populus tomentosa*) and the triploid *Eucommia* (*Eucommia ulmoides*), as well as triploid cassava—exhibit enhanced vigor and increased biomass or crop yield per plant [22, 201]. It is imperative and advisable to note that, due to the high heterozygosity of cassava as a diploid species, the development of innovative varieties through diploid hybrid breeding is exceedingly challenging, akin to the breeding history of corn [6]. Triploid breeding stands as a critically important strategy for cultivating new breakthrough varieties at present, pending the establishment of haploid systems of cassava.

The emergence of powerful gene editing technologies such as CRISPR/Cas9 is a beacon of hope, offering the potential for precise genetic modifications. While the prospects of genetic engineering and gene editing to enhance cassava traits are immense, several hurdles must be navigated. Access to comprehensive genomic resources is essential for designing specific guide RNA (gRNA) sequences that direct Cas nucleases to targeted genomic locations. Cassava, with its complex and highly heterozygous genome, often presents a challenge when compared to other crops with more accessible high-quality genomic data. This complexity can impede the application of genetic modifications. However, the ability to precisely manipulate the cassava genome and select for desired traits could significantly enhance the crop’s resilience against biotic and abiotic stresses, leading to improved productivity and nutritional value. As genomic resources continue to expand, cassava has the potential to become a model crop for the application of genomics in plant breeding. Exploring in deep the genetic resources and regulatory networks of cassava, a tropical crop with high light, high temperature adaptation, high nutritional efficiency and high biomass, is also of strategic significance for addressing the challenge of global climate change by increasing the yield and stability of major food crops in the postgenomic era.

## Supplementary Material

Web_Material_uhae341

## Data Availability

There are no new data associated with this article.
