# Supplementary material for: Advancements and strategies of genetic improvement in cassava (Manihot esculenta Crantz): from conventional to genomic approaches
Source: Hortic Res. 2024 Dec 2;12(3):uhae341. doi: 10.1093/hr/uhae341 (PMC11886850; doi:10.1093/hr/uhae341)
Supplement: Web_Material_uhae341 [file web_material_uhae341.zip › Supplementary Data Table 1.docx]

**Supplementary Data Table 1**. Key genes underlying important traits of cassava.

| **Gene name** | **Trait** | **Annotation** | **Function** | **Reference** |
| --- | --- | --- | --- | --- |
| *MePSY2* | Carotenoids content | Phytoene synthase | Promotes β-carotene accumulation | [56] |
| *MeSBE1* | Starch quality and content | Starch branch enzyme | Promotes chain-length distribution of amylopectin | [86] |
| *MeSBE2* | Starch quality and content | Starch branch enzyme | Promotes chain-length distribution of amylopectin | [86, 96] |
| *MeGBSSI* | Starch quality and content | Granule-bound starch synthase | Promotes amylose starch accumulation | [93] |
| *MePTST1* | Starch quality and content | Protein targeting to starch | Promotes GBSS localization on starch granules |  |
| *MeCWINV3* | Starch quality and content | Cell wall invertase | Inhibites sugar export from leaves to storage roots | [110] |
| *MeAPL3* | Starch content, DMC, and PPD delay | ADP-glucose pyrophosphorylase large subunit | Unknown | [111] |
| *EPSPS* | Herbicide sensitive | 1. enolpyruvylshikimate-3-   phosphate synthase | Promotes aromatic amino acids biosynthesis | [94] |
| *MeCHS3* | PPD delay | chalcone synthase | Promotes flavonoid accumulation | [119] |
| *MeANR* | PPD delay | anthocyanidin reductase | Promotes flavonoid accumulation |  |
| *MePOD12* | PPD delay | Peroxidase | Promotes ROS scavenging and lignin accumulation | [121] |
| *MeCAT1+*  *MeCu/ZnSOD* | PPD delay | Catalase,  Copper/zinc superoxide dismutase | Promotes ROS scavenging | [122] |
| *MeCAT1+*  *MeCu/ZnSOD* | Drought and cold stresses resistance | Catalase,  Copper/zinc superoxide dismutase | Promotes ROS scavenging | [163] |
| *HNL* | Protein content | Hydroxynitrile lyase | Promotes the decomposition of acetone cyanol | [133] |
| *HNL* | CG content | Hydroxynitrile lyase | Promotes the decomposition of acetone cyanol |  |
| *CYP79D1* | CG content | Cytochrome P450 | Hydroxylates valine and to a lesser extent isoleucine to form the N-hydroxyl derivative | [95, 151] |
| *CYP79D2* | CG content | Cytochrome P450 | Hydroxylates valine and to a lesser extent isoleucine to form the N-hydroxyl derivative |  |
| *CYP71E* | CG content | Cytochrome P450 | Converts oximes into cyanohydrins with a preference for oximes derived from valine | [152] |
| *UGT85K4* | CG content | UDP-glucosyl transferase | Transfers glucose to acetone cyanohydrin, producing linamarin | [153] |
| *UGT85K5* | CG content | UDP-glucosyl transferase | Transfers glucose to acetone cyanohydrin, producing linamarin |  |
| *MeGRX3* | Drought resistance | CC-type glutaredoxin | Suppresses ABA-induced stomatal closure | [81] |
| *IPT2* | Drought resistance | Isopentenyl transferase | Extendes leaf life | [161] |
| *MeRAV5* | Drought resistance | AP2 transcriptional factor | Promotes lignin accumulation and suppresses H_2_O_2_ accumulation | [162] |
| *MeMYB2* | Drought sensitive | MYB transcriptional factor | Regulates ABA-dependent pathway | [85] |
| *MeMYB2* | Cold sensitive | MYB transcriptional factor | Promotes anthocyanin accumulation | [172] |
| *MeWRKY20* | Drought resistance | WRKY transcriptional factor | Promotes ABA accumulation | [166] |
| *MeHSP90.9* | Drought resistance | Heat shock protein | Promotes ABA accumulation and suppresses H_2_O_2_ accumulation |  |
| *MeCIPK23* | Drought resistance | Calcineurin B-like proteins interacting protein kinase | Promotes ABA accumulation | [167] |
| *MeWHY1/2/3* | Drought resistance | Whirly transcriptional factor | Promotes ABA accumulation |  |
| *DIR* | Drought resistance | Long non-coding RNA | Promotes proline accumulation | [168] |
| *MeSPL9* | Drought sensitive | SQUAMOSA promoter binding protein-like | Suppresses anthocyanin, proline, soluble sugar, and JA accumulation | [170] |
| *MeMYB108* | Drought resistance | MYB transcriptional factor | Reduces leaf abscission rate | [171] |
| *MeCBF1* | Cold resistance | CBF transcriptional factor | Activates endogenous cold responsive genes | [83] |
| *MeAPX2+*  *MeCu/ZnSOD* | Cold resistance | Ascorbate peroxidase,  Copper/zinc superoxide dismutase | Enhances ROS scavenging | [164] |
| *MeTCP4* | Cold resistance | TEOSINTE BRANCHED/CYCLOIDEA/  PCF | Enhances ROS scavenging | [165] |
| *CRIR1* | Cold resistance | Cold-responsive intergenic lncRNA | Promotes proline accumulation and suppresses MDA accumulation | [169] |
| *MebHLH18* | Cold resistance | bHLH transcriptional factor | Decreases low temperature-induced leaf abscission | [173] |
| *nCBP-1* | CBSD sensitive | eIF4E isoform: novel cap-binding protein | Promotes the initiation of cap-dependent mRNA translation | [90] |
| *nCBP-2* | CBSD sensitive | eIF4E isoform: novel cap-binding protein | Promotes the initiation of cap-dependent mRNA translation |  |
| *MePOLD1* | CMD2 resistance | DNA polymerase δ subunit 1 | Mutations in MePOLD1 may introduce replication errors in geminiviruses | [185] |
| *MeSWEET10a* | CBB sensitive | Sugar transporter | Promotes pathogen hijacking of cassava sucrose | [91] |
| *MeSWEET10a* | CBB sensitive | Sugar transporter | Promotes pathogen hijacking of cassava sucrose | [92] |
| *MeWRKY79* | CBB resistance | WRKY transcriptional factor | Promotes melatonin accumulation | [189] |
| *MeHsf20* | CBB resistance | Heat shock protein | Promotes melatonin accumulation |  |
| *MeASMT2* | CBB resistance | Melatonin synthetase | Promotes melatonin accumulation |  |
| *MeHsfs3* | CBB resistance | Heat stress transcriptional factor | Promotes SA accumulation | [190] |
| *MeCAMTA3* | CBB sensitive | Calmodulin-binding transcription activators | Regulates endogenous SA and ROS accumulation | [191] |
| *MeHSP90.9* | CBB resistance | Heat shock protein | Triggers autophagy signaling | [192] |
| *MeHSP90.9* | CBB resistance | Heat shock protein | Activates SA biosynthesis pathway | [193] |
| *MeLRR1/2/3/4* | CBB resistance | NBS-LRR protein | Promotes SA and ROS accumulation | [194] |
| *MeDNAJA2* | CBB resistance | DnaJ heat shock protein | Promotes SA accumulation | [195] |
| *MeHAM1* | CBB resistance | Histone acetyltransferases | Promotes SA accumulation |  |
